# Zebrafish *atoh8* mutants do not recapitulate morpholino phenotypes

**DOI:** 10.1371/journal.pone.0171143

**Published:** 2017-02-09

**Authors:** Elsie S. Place, James C. Smith

**Affiliations:** Developmental Biology Laboratory, Francis Crick Institute, London, United Kingdom; Texas A&M University, UNITED STATES

## Abstract

Atoh8 is a bHLH transcription factor expressed in pancreas, skeletal muscle, the nervous system, and cardiovascular tissues during embryological development. Although it has been implicated in the regulation of pancreatic and endothelial cell differentiation, the phenotypic consequences of Atoh8 loss are uncertain. Conclusions from knockout studies in the mouse differ widely depending on the targeting strategy used, while *atoh8* knockdown by interfering morpholino oligonucleotides (morpholinos) in zebrafish has led to a range of developmental defects. This study characterised zebrafish embryos homozygous for *atoh8*^*sa1465*^, a loss-of-function allele of *atoh8*, in order to provide genetic evidence for the developmental role of Atoh8 in this species. Embryos homozygous for *atoh8*^*sa1465*^ present normal body morphology, swimbladder inflation, and heart looping, and survive to adulthood. These embryos do not develop pericardial oedema by 72 hpf and are not sensitised to the loss of Fog1 protein, suggesting that this previously described abnormality is not a specific phenotype. Vascular patterning and primitive haematopoiesis are unaffected in *atoh8*^*sa1465/sa1465*^ mutant embryos. Together, the data suggest that Atoh8 is dispensible for zebrafish development under standard laboratory conditions.

## Introduction

**Fig 1:**
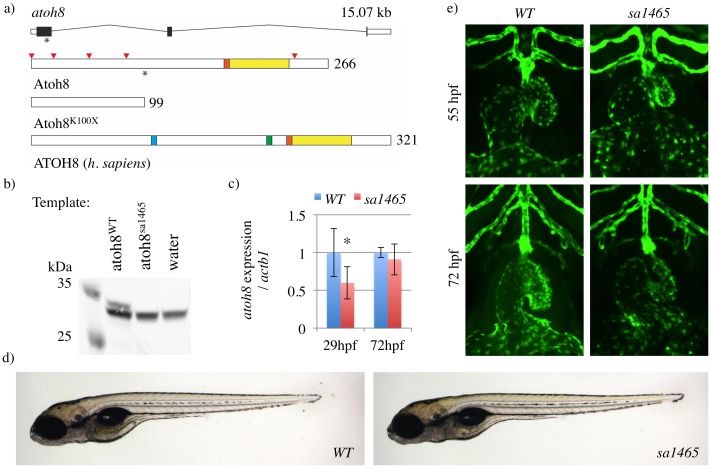
Atoh8^sa1465/sa1465^ mutants are morphologically normal, with correct heart looping. a) Structure of the zebrafish *atoh8* locus and Atoh8 protein. The positions of the A > T substitution in the atoh8^sa1465^ allele, and of Lysine 100 in Atoh8 protein, are indicated with asterisks. The red arrowheads mark the positions of each Methionine in the Atoh8 protein, and the basic (orange) and HLH (yellow) domains are indicated. The truncated protein Atoh8^K100X^ is the predicted product of atoh8^sa1465^. Human ATOH8 contains additional proline-rich (blue) and serine-rich (green) domains. b) Atoh8 Western blot on products from *in vitro* transcription/translation reactions (TNT Quick) using atoh8^WT/WT^ and atoh8^sa1465/sa1465^ as templates. Predicted size of Atoh8 = 29.8 kDa. Note that there is a strong nonspecific band at ~31 kDa. The full length membrane can be viewed in S1 Fig. c) qPCR on atoh8^WT/WT^ and atoh8^sa1465/sa1465^ embryos (hereafter labelled as '*WT*' and '*sa1465*', respectively). * *p* = 0.028. d) *WT* and *sa1465* embryos at 5 days postfertilisation, showing normal overall body morphology and swimbladder inflation. e) Confocal z-stacks showing *WT* and *sa1465* embryo heart morphology.

Atonal homologue 8 (Atoh8, also known as Math6/Hath6) is a basic helix-loop-helix protein (bHLH) implicated in neural, endocrine, and cardiovascular development. bHLH domains contains a short basic region, which mediates binding to DNA at a hexanucleotide consensus sequence (E box), and a longer HLH domain, which mediates dimerisation with other family members [1]. The Atoh8 bHLH domain is situated towards the C terminal end of the protein, and is highly conserved across vertebrates [2]. Mammalian Atoh8 also contains Proline-rich and serine-rich regions, neither of which is present in the zebrafish protein (Fig 1a) [2]. While the function of the serine-rich region is unknown, the proline-rich region appears to have weak intrinsic repressor activity [3]. However, the bHLH domain appears to be responsible for most of the regulatory activity of mammalian ATOH8 in the pancreas [3].

Atoh8 is expressed in a wide range of tissues in the embryo and adult, including the nervous system [4–6], retina [4,7], somitic muscle [6,8], kidney [5,9], heart [5,9,10], lung [5,9,10], liver [9], pancreas [3,5], blood and vascular progenitors [8,11], and vascular smooth muscle [10]. Atoh8 expression is regulated by tissue-specific bHLH genes in the pancreas, where it modulates the pro-endocrine activity of Neurog3 [5]. In endothelial cells, ATOH8 is induced by laminar shear stress, promoting endothelial fate and vascular tube assembly in hESCs [8,12]. Atoh8 has been linked to Bmp signalling in both hepatic and vascular contexts. It is induced by both BMP-9 and BMP-6 in human umbilical vein endothelial cells, and by BMP-4 in pulmonary artery smooth muscle cells. The ATOH8 promoter is bound by phosphorylated Smad1/5/8 under these conditions, suggesting direct regulation [13]. Along with *Bmp6*, *Smad7*, and *Id1*, *Atoh8* is co-regulated with the Bmp targets *Hamp1* and *Hamp2* (encoding the hepcidins) in the mouse liver [14], and ATOH8 may directly regulate *HAMP* expression at the DNA level [15]. It has furthermore been suggested that ATOH8 enhances Smad1/5/8 phosphorylation in HEK 293 cells [15].

Despite its involvement in a range of biological processes, the phenotypic consequences of loss of Atoh8 appear to be mild. Pancreatic differentiation and physiology was largely normal in embryos with conditional removal of *Atoh8* exon 1 in Pdx1-expressing pancreatic progenitors, although several lineage markers had altered expression levels, and a modest increase in somatostatin-producing (δ) cells was detected [16]. Mice with global deletion of either exon 1 or exon 2 of *Atoh8* were viable with no structural abnormalities found, notwithstanding gene expression changes in lung mesenchyme [10]. However, a genetic interaction with Gata4 was discovered, with marginally reduced survival of *Gata4*^*+/-*^*Atoh8*^*GFP/GFP*^ animals as compared to *Gata4*^*+/+*^*Atoh8*^*GFP/GFP*^ pups at postnatal day 1 (P1) and P14. These findings conflict with those of an earlier study which reported embryonic lethality in *Atoh8* null mice, with embryos exhibiting a developmental delay from around the time of gastrulation [5]. The targeting strategy in this instance involved the complete removal of exons 1 and 2 plus the intervening intron. It is possible, but unproven, that essential regulatory elements may have been removed along with the Atoh8 coding sequence, and that this may account for the vastly different consequences of the deletions in these two studies.

Discrepancies also exist concerning the published effects of *atoh8* knockdown in the zebrafish (*Danio rerio*). Gross morphological defects have been reported in zebrafish embryos injected with interfering morpholino oligonucleotides ('morpholinos') targeting *atoh8* mRNA [6]. These specimens exhibited abnormal body curvature, and defects in retinal lamination and skeletal muscle organisation. A later study reported pericardial oedema and failed swimbladder inflation in otherwise normal, morpholino-injected embryos ('morphants') [10]. Considering these inter- and intraspecific differences, and given that morpholinos can give misleading results due to interference with non-target RNAs or to toxic effects unrelated to gene knockdown [17–19], it was important to clarify the *atoh8* phenotype by genetic means. The findings of the present investigation suggest that embryos lacking Atoh8 are viable, with normal overall morphology, swimbladder inflation, heart morphology, vascular development, and primitive haematopoiesis.

## Methods

### Fish stocks, maintenance, and genotyping

All zebrafish work was carried out with approval from the Francis Crick Institute Biological Research Facility Strategic Oversight Committee and the Animal Welfare and Ethical Review Body, and in accordance with the Animals (Scientific Procedures) Act 1986, the Animal Welfare Act (2006) and the Welfare of Animals in Transport Order. Care was taken to minimize the numbers of animals used in these experiments in accordance with the ARRIVE guidelines (http://www.nc3rs.org.uk/page.asp?id=1357). Adult zebrafish were maintained and bred under standard conditions [20]. The Tg(*kdrl*:eGFP) line was obtained from Prof. Roger Patient (Weatherall Institute of Molecular Medicine, John Radcliffe Hospital, University of Oxford, Oxford, UK) [21]. The *atoh8*^*sa1465*^ allele was generated by the Sanger Centre Zebrafish Project (The Wellcome Trust Sanger Institute, Hinxton, Cambridge, UK) [22,23] and obtained from the Zebrafish International Resource Center at The University of Oregon. The *atoh8* locus was genotyped by Taqα1 digestion of a PCR fragment generated using the primers 5'-GCTCGTTTGACACTTACAGCG-3' and 5'-CCGAATCAATGTGGCCCGT-3'. The *atoh8*^*sa1465*^ line was crossed to the Tg(*kdrl*:eGFP) transgenic reporter line. The resulting progeny were in-crossed, and individuals homozygous for the wild type or mutant allele were identified by genotyping. All experiments were performed using adults from this cross, unless stated.

### Embryo microinjections and inhibitor treatments

For microinjection experiments, zebrafish embryos were injected with 0.5–2 nl morpholino solution at the 1-cell stage. LDN-193189 and DMH4 stocks were delivered in 0.1% DMSO.

### atoh8 cloning, Western blot, and TNT assay

Full length wild type atoh8 was cloned into pCRII using the primers 5'-TTTATCGATAGTCAGGCTGGACATCCGAA-3' (ClaI site underlined) and 5'-TTTCTCGAGTCTCCAGATTCACAGAAGACTTT-3' (XhoI site underlined), and cloned into the ClaI / XhoI sites of pCS2+. The sa1465 mutation was introduced by site-directed mutagenesis using the primers 5'TGCAGCAGAAGTACCTGACTCGTAATTAAGATCCGTCTCCGAAAA-3' and 5'TTTTCGGAGACGGATCTTAATTACGAGTCAGGTACTTCTGCTGCA-3' (A/T substitution underlined). For Western blotting, Rabbit anti-ATOH8 (PA5-20710, Thermo Fisher Scientific, Waltham, MA, USA) was used at 1:250, and Goat anti-Rabbit-800 (LI-COR Biosciences, Lincoln, NE, USA) was used at 1:3000. The TNT Quick SP6 assay was carried out according to the manufacturer's instructions (Promega Corporation, Madison, WI, USA).

### O-Dianisidine and peroxidase assays

Zebrafish embryos were stained in O-Dianisidine solution (0.6 mg/ml, 0.01 M sodium acetate, 0.65% H_2_O_2_, 40% (v/v) ethanol) for 15 mins in the dark, followed by post-fixing in 4% PFA. Peroxidase activity was detected using 1% Cy3-Tyramide (FP1046, Perkin-Elmer) in PBS with 0.1M imidazole and 0.03% H_2_O_2_. Embryos were fixed in advance for 1 hour, and stained for 20 minutes.

### *In situ* hybridisation and qPCR

*In situ* hybridisation was carried out according to standard procedures [24]. For the *in situ* probe, atoh8 was cloned into pCRII using the primers 5'-ATACACCAGACCACCGCAAC-3' and 5'-AAGCCAGAGCGCACATTTTT-3'. For qPCR, embryos were bisected at the level of the junction between the yolk ball and the yolk extension, and the caudal ends were collected and pooled. TRIzol^™^ (Thermo Fisher Scientific) RNA extraction and reverse transcription (M-MLV, Promega Corporation) were performed as per the manufacturer's instructions. qPCR was performed on a Roche LightCycler (Basel, Switzerland) using the following primers: *actb1* 5'- ATGAGACCACCTTCAACTCC-3', 5'- AGGAGCAAGAGAGGTGATCTCC-3', *kdrl* 5'- GCCCAGAGAGTGTGAAGACC-3', 5'- CCTCCAGCAGAACTGACTCC-3', *fli1a* 5'- AGCGCTACGCCTACAAGTTC-3', 5'- AGCTCCAGTATGGGGTTGTG-3', *sox18* 5'- GAGACGCCTACTCACCACAC-3', 5'- TCCCTCCTCAAGCACGTCTA-3', *gata1a* 5'- TTTGCCCTACACCCATCACC-3', 5'- GGTCCCGTGGATGTTTCCTT-3', *hbae3* 5'- CTGCTGGTGTCTCTGGCCATGA-3', 5'- GCAGGCTGCAGCTTTAGCGGT-3', *runx1* 5'- AGTGGACGGACCCCGAGAGC-3', 5'- ACCGCATGGCACTTCGCCTC-3', *lyz* 5'- GCACGGCCTACTGGGAAAGCA-3', 5'- CCCAGGGGTCCCGTCATCACA-3', *l-plastin* 5'- GTCGATGTGGATGGGAACGG-3', 5'- CCTCCTCGGAGTATGAGTGC-3'. Expression levels were normalised to *actb1* (beta actin).

### Imaging and statistical analysis

Imaging was performed on a Leica MZ 16F dissecting microscope and a Leica 710 confocal microscope. Confocal images were collected as z-stacks and presented as maximum intensity projections. Raw data was processed in Excel (Microsoft Corporation, Redmond, WA, USA), and statistical tests were performed using Prism 6 (Graphpad Software, Inc., San Diego, CA, USA). Bar graphs represent mean values ± standard deviations. Approximately 20 embryos were pooled for each sample for the qPCR experiment (*n* = 6). For the morpholino experiments, each data point represents between 11 and 60 embryos (*n* = 3–4). *n* numbers indicate the number of biological repeats, i.e. batches of embryos from different parents. Replicates were collected on 2–4 separate occasions. Asterisks (*) highlight *p* values of less than 0.05 (two-tailed t-tests); comparisons between *atoh8*^*WT/WT*^ and *atoh8*^*sa1465/sa1465*^ are not significant unless indicated by an asterisk. The *p* value given for the data in Table 1 was calculated using a Chi squared test.

**Table 1:** Three month survival of zebrafish from atoh8^WT/sa1465^ in-cross. *p* = 0.54.

Genotype	Observed, number	Observed, %	Expected, %
*atoh8*^*WT/WT*^	21	30	25
*atoh8*^*WT/sa1465*^	29	41	50
*atoh8*^*sa1465/sa1465*^	20	29	25
Total	70		

## Results and discussion

The *atoh8*^*sa1465*^ allele was generated in the Zebrafish Project by ENU mutagenesis of males [22,23]. This allele contains an A > T substitution which introduces a premature stop codon at the position of Lysine 100. The predicted product of this mutant allele is therefore a truncated protein, Atoh8^K100X^, which lacks the entire bHLH domain (Fig 1a). Because Atoh8 contains Methionines only at positions 1, 22, 50, 86, and 242, there are no alternative translational start sites between the K100X mutation and the end of the bHLH domain.

To confirm the predicted effect of this mutation, the full coding sequence of zebrafish *atoh8* was cloned into pCS2+, and the *sa1465* mutation was introduced by site-directed mutagenesis. Both constructs were used as templates in an *in vitro* transcription-translation reaction (TNT Quick assay), and the reaction products were assessed by Western blot. An antibody directed against a C-terminal region of human ATOH8 (94% homology to zebrafish Atoh8) failed to detect any product arising from the mutated form (Fig 1b). qPCR was performed on *atoh8*^*WT/WT*^ and *atoh8*^*sa1465/sa1465*^ embryos to assess whether *atoh8* transcript was lost due to nonsense-mediated decay. Although transcript levels were reduced by 40% at 29 hours postfertilisation (hpf), at 72 hpf *atoh8* mRNA levels were unchanged (Fig 1c). Thus, although nonsense-mediated decay of *atoh8*^*sa1465*^ transcripts appears to be minimal, this allele codes for a protein lacking the main functional domain of Atoh8, and as such probably represents a severe loss-of-function mutation.

Surprisingly, considering the published *atoh8* morphant phenotypes, *atoh8*^*sa1465/sa1465*^ embryos were viable and survived to adulthood at Mendelian ratios (Table 1). These embryos had a straight body axis and no overt morphological abnormalities. Their eyes and somite boundaries appeared normal, and their swimbladders inflated by 5 days postfertilisation (dpf) (Fig 1d). No pericardial oedema was seen at any timepoint, and their hearts looped correctly (Fig 1e). Therefore, none of the defects previously described in zebrafish *atoh8* morphants were observed in *atoh8*^*sa1465/sa1465*^ mutant embryos.

**Fig 2:**
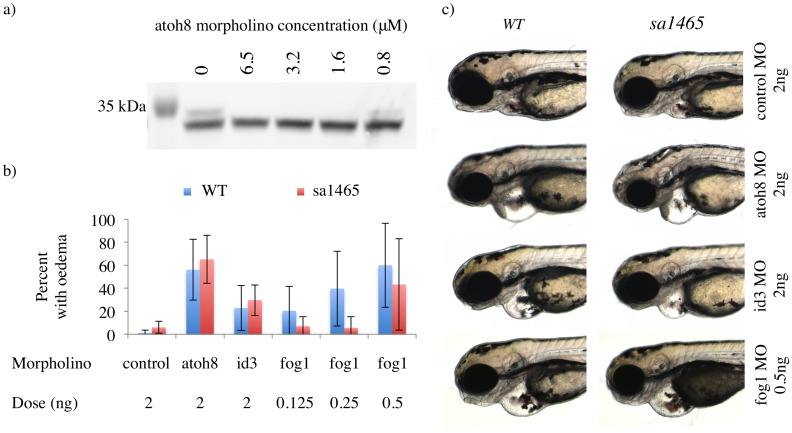
No alteration in response to atoh8 or fog1 knockdown in atoh8^sa1465/sa1465^ mutants. a) Atoh8 Western blot on products from *in vitro* transcription/translation reactions (TNT Quick) seeded with atoh8 morpholino at the indicated concentrations. The full length membrane can be viewed in S2 Fig. b) Percentage of *WT* and *sa1465* embryos displaying pericardial oedema at 72 hpf, following injection with the indicated morpholinos. c) Examples of pericardial oedema in *WT* and *sa1465* embryos injected with atoh8, id3, and fog1 morpholinos. Imaged at 72 hpf.

To investigate the discrepancies between the mutant and morphant phenotypes, we obtained an ATG-blocking atoh8 morpholino, which was used in a previous publication ('MO1', [10]], and overlaps with 'MO1' from the Yao et al. study at 24 out of 25 bases [6]. This morpholino efficiently inhibited the translation of Atoh8 in the TNT Quick assay (Fig 2a). Using this morpholino, Rawnsley *et al*. described a heart defect in *atoh8* morphants that manifested as pericardial oedema by 72 hpf [10]. Confirming the result of this study, 56% of embryos injected with a low dose (2 ng) of this morpholino exhibited pericardial effusion by 72 hpf (Fig 2b and 2c). The eye, muscle, and other defects seen in the earlier morpholino-based study were not observed [6].

Why should the effects of *atoh8* knockout and knockdown differ? It is well known that morpholinos can induce toxic and off-target effects that can prove misleading [17–19]. However, it has also been proposed that morpholino knockdown of a protein may in some cases provoke a more severe phenotype than a genetic knockout, due to compensatory mechanisms operating in mutants but not in morphants [25]. Where mutants present a milder phenotype (or none) than morphants, a genuine biological basis for the discrepancy can be ruled out by injecting the morpholino into mutant embryos. Because the embryo already lacks the targeted protein, any 'phenotype' observed cannot result from the loss of this protein. Conversely, if the morphant phenotype does *not* manifest in mutant embryos, then one possible interpretation is that genetic compensation protects mutants from the effects of protein loss.

In fact, the incidence of oedema in *atoh8*^*sa1465/sa1465*^ embryos injected with the atoh8 morpholino was similar to that seen in *atoh8*^*WT/WT*^ embryos, strongly suggesting that the oedema phenotype is not a specific effect of Atoh8 protein loss. However, since Atoh8 protein is absent in both injected groups, the possibility remains that *atoh8*^*sa1465/sa1465*^ mutants have a subtle underlying cardiovascular defect that renders them more vulnerable to physiological insults than wild type embryos. This would be analagous to the situation in *id4* mutant embryos, which develop normal hearts under standard conditions, but exhibit retrograde blood flow through the heart when challenged with increased cardiac output [26]. To exclude this possibility, an id3 morpholino was injected into *atoh8*^*WT/WT*^ and *atoh8*^*sa1465/sa1465*^ embryos. This morpholino induced oedema in a minority of *atoh8*^*WT/WT*^ embryos (23%) when injected at the same dose as the *atoh8* morpholino (2 ng). No significant difference was seen in rates of oedema between wild type and mutant embryos, suggesting that *atoh8*^*sa1465/sa1465*^ mutants are not more susceptible to developing pericardial oedema than their wild type siblings.

Finally, we investigated the putative genetic interaction between Atoh8 and Fog1 in the heart. The FOG family of proteins regulate GATA factors in diverse tissues including heart, blood, and liver [27–30]. Rawnsley *et al*., demonstrated a physical interaction between Atoh8, Fog1, and Gata4, with Fog1 bridging the indirect interaction between Atoh8 and Gata4 [10]. In careful work, they also showed that low doses of morpholinos against two or three of these targets synergistically induced the pericardial oedema phenotype. However, the incidence of oedema in *fog1* morphants was not increased in *atoh8*^*sa1465/sa1465*^ embryos compared to wild type (Fig 2b and 2c). Therefore it seems very unlikely that that pericardial oedema consistently observed in *atoh8* morphants represents a true phenotype.

**Fig 3:**
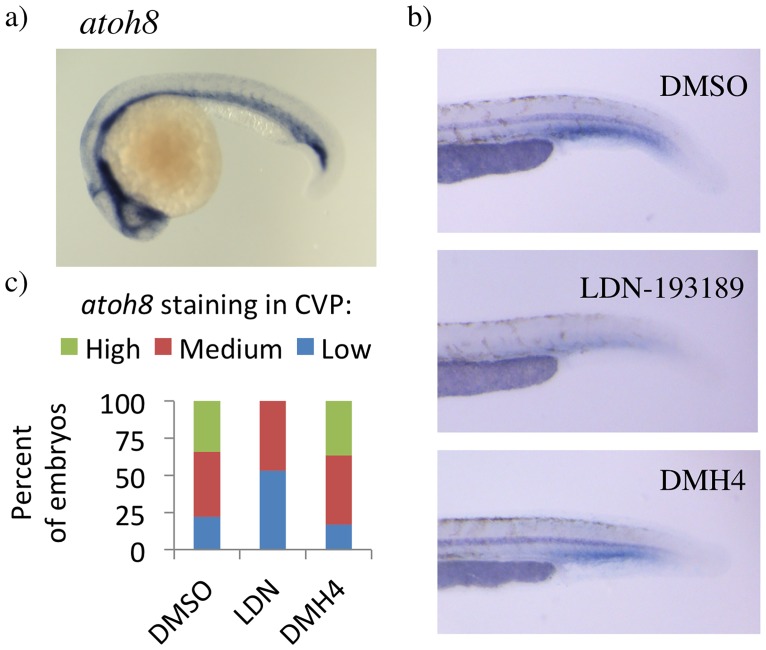
Vascular expression of *atoh8*, and regulation by Bmps. a) *atoh8* expression in 24 hpf Tg(*kdrl*:eGFP) embryos. b) *atoh8* expression in the blood island of 29 hpf embryos. c) Levels of *atoh8* staining in caudal vein plexus (CVP) region of 29 hpf zebrafish embryos. Tg(*kdrl*:eGFP) embryos treated with DMSO, 5 μM LDN-193189, or 10 μM DMH4. Number of embryos: 50 (DMSO), 66 (LDN-193189), 30 (DMH4).

**Fig 4:**
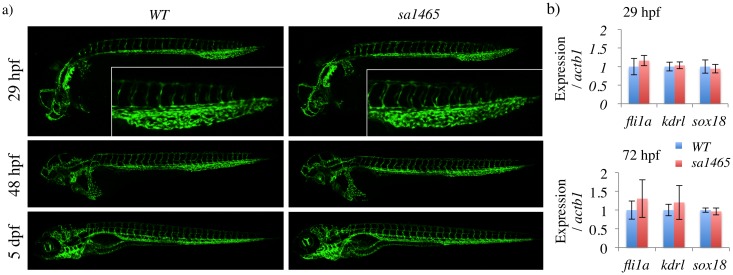
*sa1465* embryos have normal vascular patterning and caudal vein plexus formation. a) Confocal z-stacks of WT and sa1465 embryos at the indicated stages. Insets (top) = caudal vein plexus region of 29 hpf embryos. b) qPCR for vascular markers in *WT* and *sa1465* embryos at 29 and 72 hpf.

If *atoh8*^*sa1465/sa1465*^ mutants do not have retina, skeletal muscle, heart, or swimbladder defects, then what is the *atoh8* loss-of-function phenotype? Several reports link ATOH8 to vascular biology and to the BMP signalling pathway [8,12–15]. Consistent with previous work [8], zebrafish *atoh8* is strongly expressed in vascular tissues at 24 hpf (Fig 3a). Furthermore, *atoh8* expression at 29 hpf in the blood island of the tail was modestly reduced with the Bmp signalling inhibitor LDN-193189, but not with the Vegf inhibitor DMH4 (Fig 3b and 3c). This is a site of active angiogenesis at this timepoint, with Bmp signalling driving the formation of the caudal vein plexus (CVP) from the caudal vein [31,32]. Vascular development was therefore assessed in *atoh8*^*sa1465/sa1465*^ mutants. No abnormalities were noted in mutant embryos at any timepoint up to 5 dpf, and the CVP formed normally (Fig 4).

Bearing in mind the links between ATOH8 and Bmp signalling, the influence of *atoh8* on CVP formation was investigated further. Atoh8 appears to function in the pancreas by competitively inhibiting gene activation by Neurog3/E47 dimers [3]. Although Atoh8 has a basic (DNA-binding) domain and weak intrinsic repressor activity deriving from its Proline-rich domain, this inhibition appears to operate partly by sequestration [3]. In this respect there is mechanistic overlap with the Id proteins, canonical Bmp targets essential for normal vascular development [33,34]. The possibility that Atoh8 may function redundantly with Id proteins was investigated by comparing the effects of Id knockdown in wild type and mutant embryos. A combination of morpholinos against *id1*, *id2a*, and *id3* was injected into *atoh8*^*WT/WT*^ and *atoh8*^*sa1465/sa1465*^ embryos, plus a *tp53* morpholino to abrogate toxicity [17]. No differences were noted in CVP formation between control and mutants. The Id1 morpholino was also tested in isolation at a higher dose, because *Id1* is strongly expressed in the axial vasculature [35], but still, no angiogenesis phenotype was seen (S3 Fig).

Since blood and endothelial cells arise from common precursors [36], embryonic haematopoiesis was also investigated in *atoh8*^*sa1465/sa1465*^ mutant embryos. Phylogenetically, ATOH8 has been placed close to the haemangioblast regulators SCL and TAL1 [37]. Furthermore, the ability of Atoh8 to bind Fog1 [10], combined with the strikingly similar 24 hpf expression patterns of these two factors (Fig 3a) [11,27,38], suggests a possible role for Atoh8 in haematopoiesis. In addition to its role in heart looping, Fog1 cooperates with GATA1 and interacts with NuRD in erythrocyte and megakaryocyte development [28,30,39,40], and morpholino knockdown of *fog1* in zebrafish promotes myelopoiesis at the expense of erythropoiesis [11,27].

**Fig 5:**
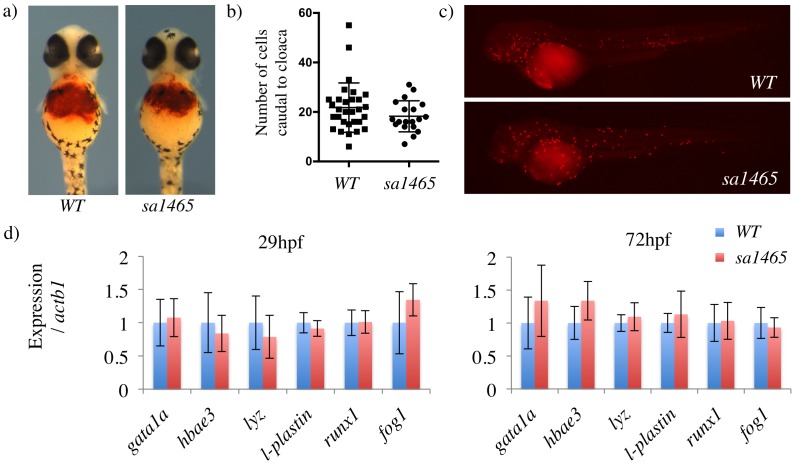
Normal primitive haematopoiesis in *sa1465* embryos. a) O-dianisidine staining in 48 hpf *WT* and *sa1465* embryos. b) Number of peroxidase-positive cells present in the distal tail (caudal to the cloaca), in 48 hpf *WT* and *sa1465* embryos. c) Examples of peroxidase staining in 48 hpf *WT* and *sa1465* embryos. d) qPCR for blood markers in *WT* and *sa1465* embryos.

O-dianisidine staining of 48 hpf *atoh8*^*WT/WT*^ and *atoh8*^*sa1465/sa1465*^ embryos revealed similar levels of circulating erythrocytes in each (Fig 5a). An assay for peroxidase activity suggested that normal numbers of myeloid cells were present in *atoh8*^*sa1465/sa1465*^ mutants (Fig 5b), and a panel of blood markers were unchanged, suggesting that primitive haematopoiesis is unaffected in embryos lacking Atoh8 (Fig 5c). *Fog1* and the haematopoietic stem cell transcription factor *runx1* were also expressed at normal levels. In summary, zebrafish *atoh8*^*sa1465/sa1465*^ mutants show none of the defects described in *atoh8* morphants, and vascular development and primitive haematopoiesis appear to proceed normally in these embryos.

This study aimed to clarify the role of Atoh8 in zebrafish development, using a genetic mutant not subject to the pitfalls of morpholino artefacts. No overt abnormalities were noted in *atoh8*^*sa1465/sa1465*^ mutant embryos, making it unlikely that loss of Atoh8 results in retinal, skeletal muscle, cardiac or swimbladder defects. Although genetic mutants are usually considered superior to morphants as models for protein deficiency, the possibility of residual protein function can arise due to alternative splicing, alternative initiation codons, translational read-through, or unanticipated functions of truncated proteins [19,41]. Owing to the position of the premature stop codon in the *atoh8*^*sa1465*^ allele with respect to the bHLH domain, potential downstream initiation codons, and exon boundaries, it is likely that this mutation represents a severe loss-of-function mutation and is therefore an excellent model for Atoh8 deficiency. However, in the absence of a frameshift, it is possible that some full-length protein may still be produced due to translation read-through.

We have presented evidence that the pericardial oedema seen in atoh8 morphants may be an artefact, despite the apparent specificity of this phenotype. The fact that low rates of oedema were observed with an id3 morpholino underscores that this is a common consequence of morpholino injection, and indeed of physiological insults more generally. One study reported that pericardial oedema similar to that observed here resulted from five different stressors, acting through diverse mechanisms [42]. Reduced cardiomyocyte proliferation and pericardial effusion therefore appear to be the ultimate outcome of a wide range of specific and nonspecific physiological challenges. The possibility that *atoh8*^*sa1465/sa1465*^ embryos may be more susceptible than wild type embryos to such stressors was investigated, but appears not to be the case.

## Conclusion

We conclude that zebrafish embryos lacking Atoh8 are grossly normal, presenting normal circulation and swimbladder inflation. Our data suggests that Atoh8 is dispensible in zebrafish under standard laboratory conditions, although the possibility remains of subtle and hitherto undetected effects on embryological development and adult physiology.

## Supporting information

S1 Fig Membrane shown in Fig 1b, full length. (TIF)

S2 Fig Membrane shown in Fig 2b, full length. (TIF)

S3 Fig No difference in caudal vein plexus formation between *WT* and *sa1465* embryos injected with *id* morpholinos. Morpholinos and doses as indicated. Imaged at 32 hpf.
(TIF)

S1 Table qPCR spreadsheet for Fig 1c. (XLSX)

S2 Table Full data for Fig 2b. (XLSX)

S3 Table qPCR spreadsheet for Figs 4b and 5d. (XLSX)
