# Correction: Zebrafish *atoh8* mutants do not recapitulate morpholino phenotypes

**DOI:** 10.1371/journal.pone.0175608

**Published:** 2017-04-06

**Authors:** Elsie S. Place, James C. Smith

The sequences for several morpholino oligonucleotides used in the study are not included in the published article. The morpholino sequences are as follows:

atoh8 5'-GTTTAGATGTGGGTTCTTCATTTCG-3', id1 5'-CGCAGGTAGGTCCCACAACTTTCAT-3', id2a 5'-GCCTTCATGTTGACAGCAGGATTTC-3', id3 5'-CGAACGGGACTGATCGCCTTCATTC-3', fog1 5'-TCATGTCCCCCTTACCTCACTGGCA-3', tp53 5'-GCGCCATTGCTTTGCAAGAATTG-3'.
