# Supplementary figures and images for: Zebrafish atoh8 mutants do not recapitulate morpholino phenotypes
Source: PLoS One. 2017 Feb 9;12(2):e0171143. doi: 10.1371/journal.pone.0171143 (PMC5300237; doi:10.1371/journal.pone.0171143)

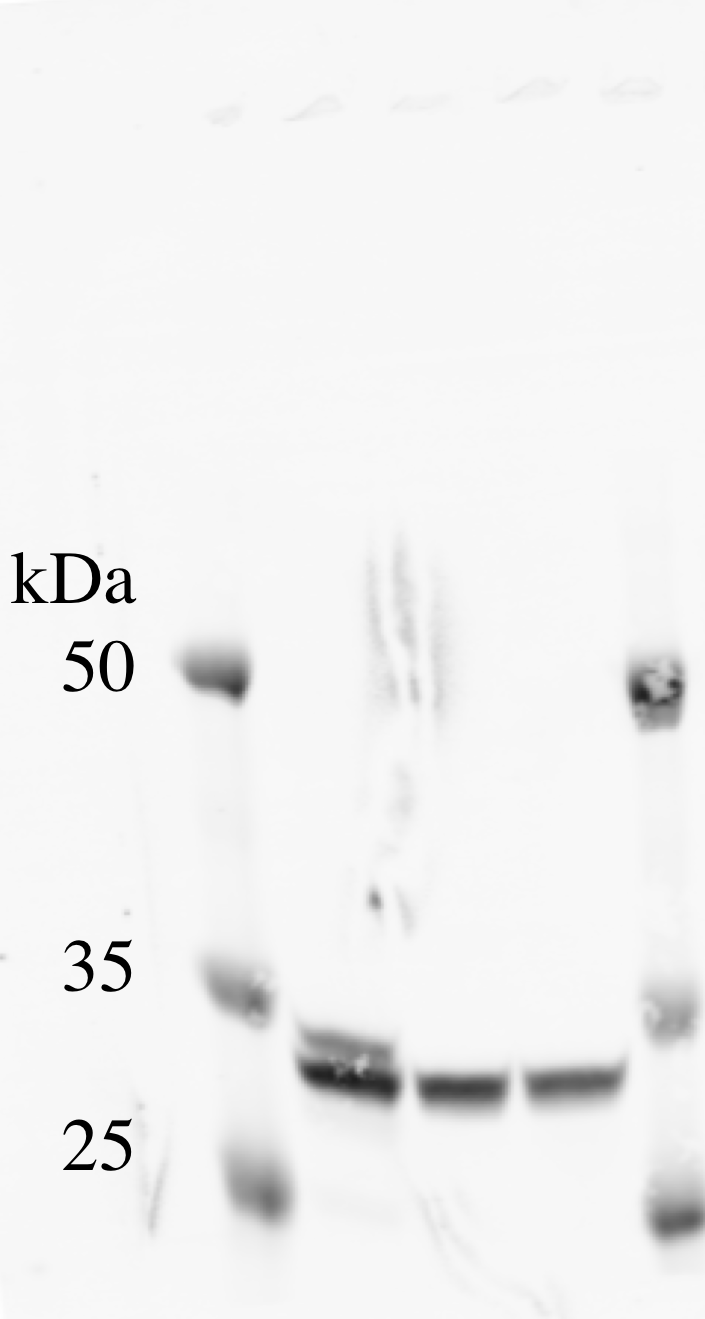

Supplement: S1 Fig — (TIF) [file pone.0171143.s001.tif]

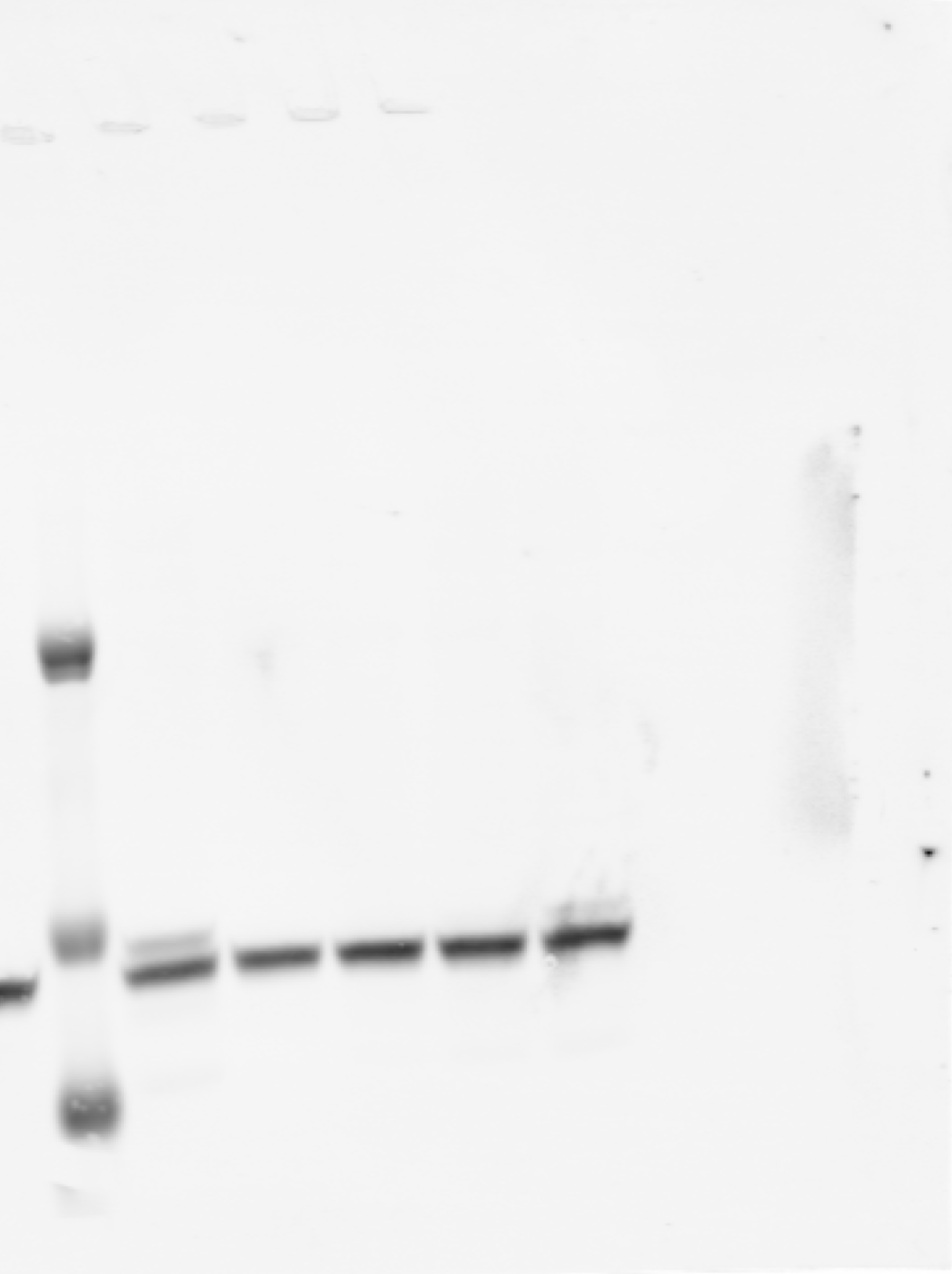

Supplement: S2 Fig — (TIF) [file pone.0171143.s002.tif]

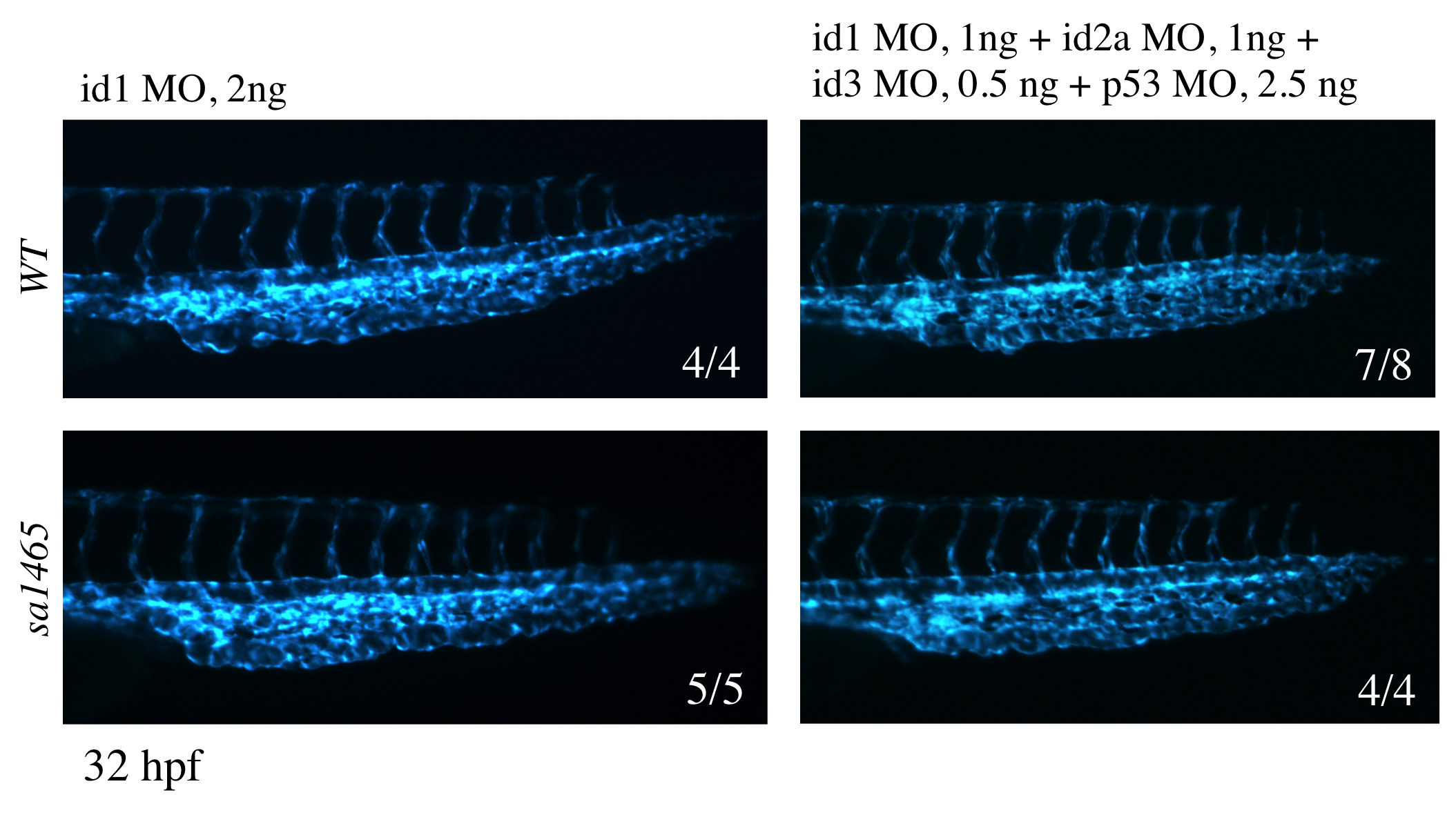

Supplement: S3 Fig — Morpholinos and doses as indicated. Imaged at 32 hpf. (TIF) [file pone.0171143.s003.tif]
